# VernoniasubgenusAustrovernonia, a new subgenus from South America (Compositae, Vernonieae, Vernoniinae)

**DOI:** 10.3897/phytokeys.110.28890

**Published:** 2018-11-05

**Authors:** Harold Robinson, Vicki A. Funk

**Affiliations:** 1 Department of Botany, MRC 166, NMNH, P.O. Box 37012, Smithsonian Institution, Washington, D.C. 20013-7012, USA Smithsonian Institution Washington United States of America

**Keywords:** Asteraceae, corolla ducts, DNA GenBank, lectotype, North America, semi-aquatic plants, South America, *Vernonia, Vernonia* subg. *Austrovernonia*

## Abstract

*Vernoniaincana* Less. and *V.echioides* Less. are two semi-aquatic species from southern South American that are referred to as “the semi-aquatic *Vernonia* of South America” and they have been, until now, retained as members of *Vernonia* mostly because each had some unusual characters that made them hard to place. Based on an ongoing molecular study, we can now say that *V.incana* is the sister taxon to all true *Vernonia* and can therefore be responsibly left in *Vernonia* but, because of its morphological and geographic differences, it is now assigned to its own subgenus: *Vernonia* subg. *Austrovernonia. Vernoniaechioides* is not closely related to *V.incana* and is now part of a separate investigation. This placement of *V.incana* as sister to the true *Vernonia* species brings up the possibility of a South American root for the *Vernonia* clade and perhaps indicates a propensity in the lineage for growing in or near water. The species *V.incana* is lectotypified.

## Introduction

Revisions of the concept of *Vernonia* Schreb. during the last 30 years, as summarised in the treatments of American Vernonieae (Robinson 1999b; also see discussion on global distribution in Keeley and Robinson 2009), have excluded the non-North American species once placed in the genus. The genus *Vernonia* Schreb. currently contains 20 species, 17 are native to eastern and central North America (Strother 2006), three of which cross the border into Coahuila, Mexico (Table 1). Additionally, there is one species in Eastern Mexico, *V.greggii* A.Gray with 2–3 varieties and two from South America: *V.echioides* Less. and *V.incana* Less. (Table 1). The South American species share several features with other members of typical *Vernonia*: rhizomiform rootstocks, totally cymiform inflorescences and sublophate pollen (shared by all members of the subtribe Vernoniinae). Due to the shared habits and similarity in pollen, there was no reason to remove the two species from *Vernonia*. However, the question of where to place these two somewhat odd South American species continued to puzzle students of the tribe. The situation remained unchanged as attention turned to the greater problems found in the Old World Vernonieae (Robinson 1999a, Robinson et al. 2016, Bunwong et al. 2014, Funk and Robinson 2017).

**Table 1.** Species of true *Vernonia*. Names in bold indicate that they have been sequenced. Two species, *V.arkansana* and *V.lettermannii* have recently been collected and will be added to future sequencing efforts.

Species	Locality
*Vernonia* in USA (Robinson 1999; Strother 2006)	
*V.acaulis* (Walter) Gleason	Carolinas & Georgia
***V.angustifolia* Michx.**	Southeast US
*V.arkansana* DC.	Central US
***V.baldwinii* Torr.**	Central US
***V.blodgettii* Small**	Florida & the Bahamas
***V.fasciculata* Michx.**	Central US
*V.flaccidifolia* Small	Southeast US
***V.gigantea* (Walter) Trel.**	Eastern US
***V.glauca* (L.) Willd.**	Eastern US
*V.larseniae* B.L.King & S.B.Jones	Texas & Coahuila Mexico
*V.lettermannii* Engelm. ex A.Gray	Arkansas & Oklahoma
***V.lindheimeri* A.Gray & Engelm.**	Texas & Coahuila Mexico
***V.missurica* Raf.**	Central US
***V.marginata* (Torr.) Raf.**	Southcentral US & Coahuila Mexico
***V.noveboracensis* (L.) Michx.**	Eastern US
***V.texana* (A.Gray) Small**	Southcentral US
*V.pulchella* Small	Georgia
*Vernonia* only in Mexico (Turner 2007)	
***V.greggii* A.Gray**	Mexico, Sierra Madre Oriental
*V.ervendbergii* A.Gray [= *V.greggii*]	
V.greggiivar.schaffneri A.Gray [previously *V.schaffneri* A.Gray]	
*Vernonia* in South America	
***V.incana* Less.**	Argentina, southern Brazil, Uruguay
***V.echioides* Less.**	Argentina, southern Brazil, Uruguay

Recently, this relationship was called into question as a result of a study of corolla lobe anatomy in which a brief observation of the two species was included (Robinson and Yankowski 2016). The corolla lobe feature of “multiple longitudinal ducts” was first noted in the description of *Trepadonia* H.Rob. (1994) and was recognised in the Robinson (1999b) treatment, as being characteristic of the typical element of the Vernonieae. The feature was found in the corolla lobes of *Vernonia, Vernonanthura* H.Rob. and *Trepadonia* H. Rob. and was illustrated schematically in the Robinson (1999a) treatment and with photographs in the Robinson and Yankowski (2016) treatment. The feature was seen as a potential defining characteristic for the *Vernonia* typical group. However, in the study by Robinson and Yankowski (2016), the corolla lobes were examined in full anatomical detail and in only that study were the corolla lobes of the two South American species examined for the characteristic ducts. *Vernoniaechioides* and *V.incana*, as seen at that time, did not show the multiple longitudinal ducts. Thus, on the basis of the remote geographical location, details cited below and the apparent lack of multiple ducts in the species, it was suggested that the species are not closely related to *Vernonia* and needed further study.

**Figure 1.**
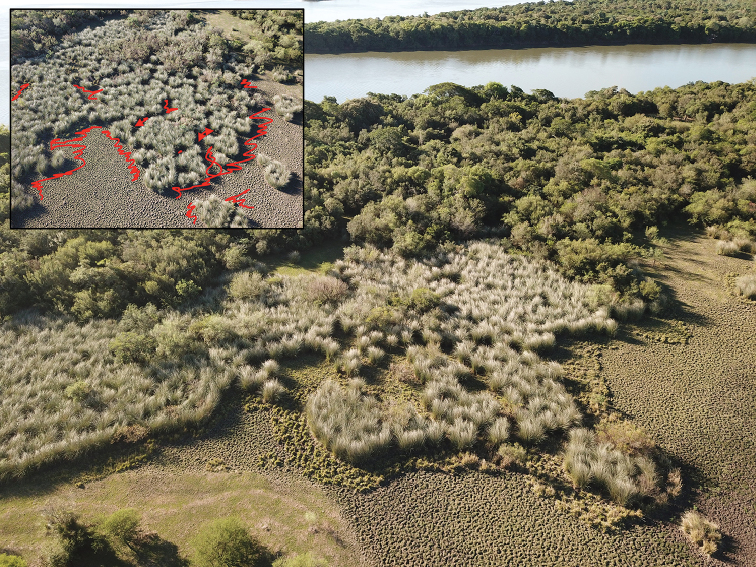
Habitat of *Vernoniaincana* and *V.echioides* taken with a drone at the Environmental Protection Area *Rincon de Franquía*, Cuareim River, Artigas, Uruguay: insert shows in red the areas where the semi-aquatic *Vernonias* of South America can be found. (photos by J.M. Bonifacino, MVFA).

*Vernoniaincana* and *V.echioides* inhabit hydromorphic soils always growing along river banks in open patches of *Coleataeniaprionitis* (Nees) Soreng and other grasses forming tall grass associations locally known as “pajonales” (Fig. 1; JM Bonifacino, pers. comm.). The soil in which they grow is sufficiently soft that the roots are often extracted with the plants.

This study was designed to determine where in the Vernonieae to place these two semi-aquatic species. It was hoped that results of the DNA sequencing would fully resolve the issue.

## Materials and methods

We were fortunate that our colleagues Sterling Keeley, Tim Gallaher and Jason Cantley were just finishing a project that included an updated molecular phylogeny of the entire Vernonieae tribe (Keeley et al., in prep.). We were even more fortunate that they were willing to share their published (Keeley et al. 2007) and unpublished data. They did not sequence either of the semi-aquatic taxa, so, as part of this study, those two taxa were sequenced using the same molecular markers as Keeley et al. (in prep.) (*ITS1*, *ITS2*, *5pt8s*, *trnL*, *ndhf*-in part) and the sequences from these two species were combined with 125 taxa from Keeley et al. (in prep.), mostly from the Americas. Extractions were made from one specimen of *V.incana* housed in the U.S. National Herbarium (US). The *5pt8s* marker was not informative within the true *Vernonia* so its use was not continued.

### Sequencing and alignment

Samples were prepared by hand grinding leaf material with liquid N in a mortar and pestle followed by extraction using DNeasy Plant Mini Kit following the manufacturer’s protocols (Qiagen, Valencia, California, USA).

Methods followed those of Keeley et al. (2007) so that the data could be combined. The full nuclear ITS region, part of the chloroplast gene *ndhF* and the non-coding spacer region *trnL-F* were used for the analyses. Sequencing was done at the Laboratory of Analytical Biology, NMNH, Smithsonian Institution. The resulting sequences were added to the previously selected taxa from the existing nexus file generated by Keeley et al. (in prep) and aligned by eye. Preliminary phylogenetic trees were produced using RAxML (Randomized Axelerated Maximum Likelihood; Stamatakis 2014).

Two phylogenies were generated using two different versions of the dataset. As all of the taxa in the database had ITS but some of the other markers were missing for some of the taxa, especially a group of Brazilian taxa that were of particular interest, the data were analysed twice. The first analysis included all the taxa and only used the ITS data. The second analysis included just the taxa that had all (or nearly all) of the data. The placement of *Vernoniaincana* was the same in both analyses so we are confident that we have the correct sister group relationship for this taxon. However, *Vernoniaechioides* was not an immediate relative of *V.incana* and its placement was ambiguous, thus necessitating the addition of more species and it will be dealt with in a later publication.

The GenBank numbers and voucher information for *Vernoniaincana* are listed in Table 2. The numbers for the remaining taxa were either published in Keeley et al. 2007 or will be published in Keeley et al. (in prep.).

**Table 2.** Voucher information and GenBank numbers for *Vernoniaincana* Less. Voucher: Paraguay, Central: Estero del Ypoa, Villeta - Puerto Guyrati, 11 km S of Villeta, east of trail to Villa Oliva. Inundated savannah on clay soil with patches of forest, Zardini & Aquino 4305, 16 Dec 1992, housed at US [US Catalog No.: 3299682; Barcode: 01627756]. Available on line at https://collections.nmnh.si.edu/search/botany/ [search on collecting number].

Gene/gene region	GenBank numbers
ITS1&2	MH933736
*trnLF*	MH933737
*ndhF*	MH933738

## Results

**Figure 2.**
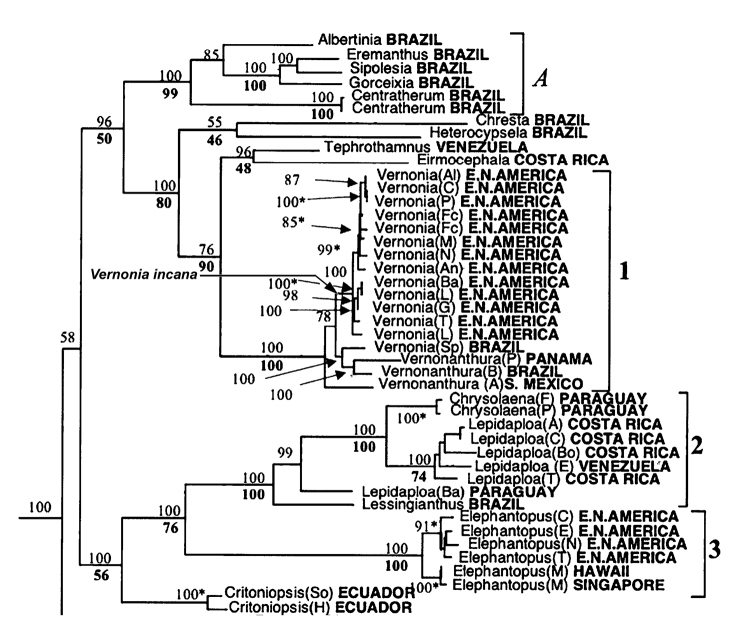
Phylogeny with an indication of where *Vernoniaincana* Less. is placed (arrow). The phylogeny is modified from Keeley et al. 2017 and does not take into account all the taxa that were in the analysis performed by the authors using data from Keeley et al. (in prep.).

The results show that while both species remain in the sub-tribe Vernoniinae, they are not in the same clade. *Vernoniaincana* is the sister group of the clade consisting of the true *Vernonia*. Although the phylogeny cannot be presented until the Keeley et al. paper is published, it is possible to visualise the placement of *V.incana* by examining the published trees of Keeley et al. (2007). In that publication (Keeley et al. 2007), the phylogenies contained 13 samples representing 11 of the 17 species of true *Vernonia*, the same ones that are used in the current Keeley et al. (in prep) article. The placement of *V.incana* in our phylogeny was in clade number 1 in Keeley et al. (2007) as the sister taxon of the group containing all of the 11 North American species. Figure 2 in this paper is a portion of one of the figures from Keeley et al. (2007) and is used to indicate where *V.incana* is placed.

The suggested placement of *V.incana* is in conflict with the findings of Robinson and Yankowski (2016) which suggested exclusion from the immediate relationship to *Vernonia*. The nearest relatives in the subtribe Vernoniinae, *Vernonias.s.*, *Vernonanthura* and *Trepadonia*, all have obvious multiple longitudinal ducts in the corolla lobes. A careful re-examination of the corolla lobes of *V.incana* confirmed that ducts were not evident under the microscope in whole mounts of the corollas. If any ducts are present, they are too obscure to be seen in this way. It is possible that the more semi-aquatic habitat of these species has somehow suppressed the production of the ducts. Thus, while being a character of some value, the ducts are not 100% reliable for defining the subtribe Vernoniinae.

Pollen morphology, that is so helpful in many other members of the Vernonieae (Bunwong and Chantaranothai 2008; Robinson and Skvarla 2010, Robinson et al. 2016), is not useful because pollen of all the genera involved here have the structure that, in the Vernonieae, is called sublophate. This sublophate pollen type has a moderately uneven distribution of spines and muri on the grains with a continuous perforated tectum covering all the non-colpate surfaces and is common in the tribe. Even the pollen sizes are alike, being 40–50 µm in diameter (in fluid).

**Figure 3.**
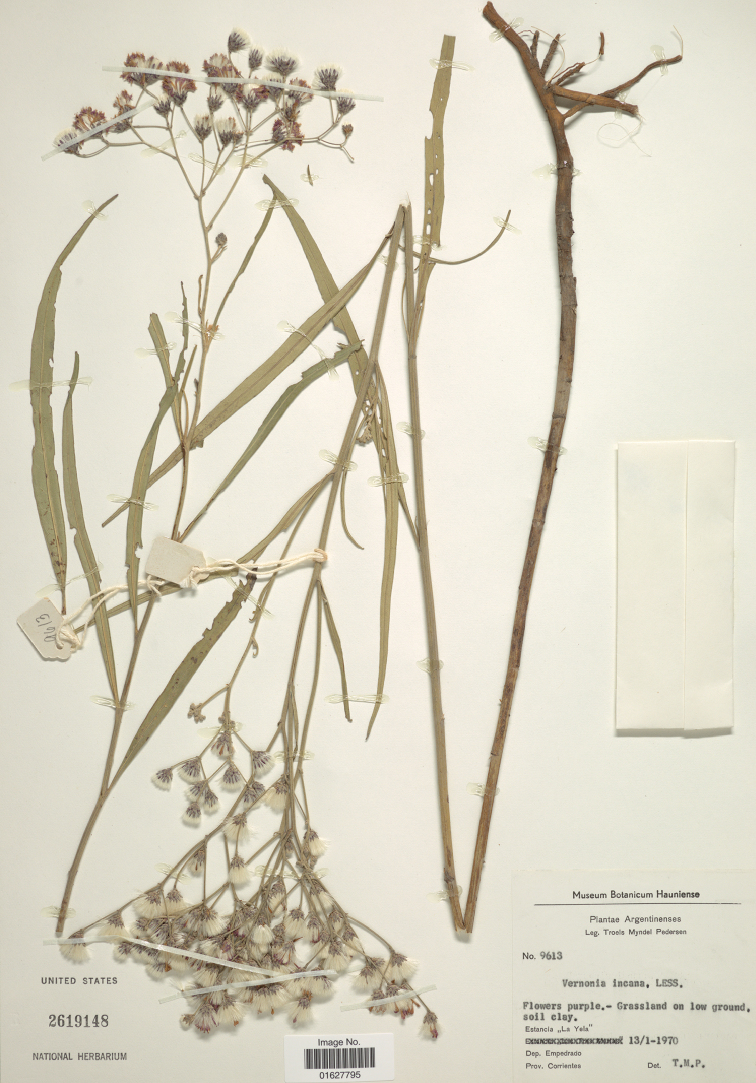
Image of US herbarium sheet of *Vernoniaincana* Less. which shows the two types of roots: fleshy and fibrous. [photo courtesy of I. Lin, US].

One of the features that the two South American species share is semi-aquatic habit. The habit is clearly stated in the label data of many of the specimens. Labels for *V.incana* cite: gallery forest, inundated savannah, flooded field, laguna margin, marsh or emerging from water. One specimen, [Mary A. Walter 118, Paraguay, Esterito, Dist. Yataity, Dept. Ñeembucú, no. 15, Jan 1975, from the Herbarium Univ. Florida Agricultural Experimental Station] has an additional label in the packet stating: “Coastal for nearly all marshes – common … leaves emerge above water level, often dense”. The rhizomes of the species have fleshy lateral roots apparently a specialisation for their habitat (Fig. 3) and are easily extracted from the soil in contrast to the North American *Vernonia* which have deep thick roots that are extremely difficult to extract. Indeed, recent fieldwork in the central USA showed that all seven species collected had large underground root systems that defied normal extraction procedures (Funk pers. comm.). The semi-aquatic habit of the South American species is especially interesting because the North American species of the true *Vernonia* are all spatially related to water in that they grow near water (river banks, near boat ramps, in boggy areas or in moist soil) but not in standing water as do many of the South American collections. This propensity to grow in or near water may indicate a *prope aquam* origin for the genus which is in contrast with the other members of the subtribe Vernoniinae: *Vernonanthura* is often in open areas requiring fire-survival strategies and *Trepadonia* is a climbing plant.

Based on the results of the DNA analysis, both of these species belong in the subtribe Vernoniinae. Of the two species, the position of *V.incana* is separate from *V.echioides* and is the sister group of *Vernonias.s.* but geographically remote from the rest of the genus, without obvious corolla lobe ducts and also without a basal node on the style. The basal node has been observed on the style in all examined species of *Vernonias.s.*, *Vernonanthura* and *Trepadonia* (Robinson 1999b, table 2). These differences between *V.incana* and its more northern relatives indicate that it is not a recent introduction from the north. It is interesting to consider whether or not this placement indicates a possible location for the origin of the *Vernonias.s.* clade: all of the related clades in the Vernoniinae are rooted in either in Meso- or South America.

The morphological differences and geographical separation support a new subgeneric position for *V.incana*.

The new subgenus is named for its geographic position with regard to typical *Vernonia* of North and Central America.

### Taxonomy

### Family: Compositae (Asteraceae)

#### Subfamily: Cichorioideae

##### Tribe: Vernonieae

###### Subtribe: Vernoniinae

####### Genus: *Vernonia*

######## Vernonia subg. Austrovernonia H.Rob. & V.A. Funk, subg. nov.

Taxon classificationPlantaeAsteralesAsteraceae

urn:lsid:ipni.org:names:77191608-1

######### Subgeneritype.

Vernoniaincana Less.

######### Diagnosis.

Semi-aquatic herbs 1.0–1.5 m tall, with base an apparently unbranched contorted taproot or rhizome, with fleshy lateral roots rather easily extracted from the soil. Stems striated, subsericeous with appressed T-shaped trichomes. Leaves alternate, sessile with narrow bases; blades linear to elliptical, margins sub-entire with sparse minute denticulations, apices narrowly acute, both surfaces with glandular dots, sub-sericeous with appressed T-shaped trichomes, abaxial surface somewhat paler; venation pinnate with usually 12 or more curving secondary veins on each side. Inflorescences cymbiform, with basal capitulum often appearing sessile as a result of 2–3 or more immediately subtending lateral branches, with branches loosely seriate-cymose. Capitula mostly appearing pedunculate as a result of lowered positions of subtending lateral branches; involucres sub-imbricate with 25–35 gradate involucral bracts in 3–5 series; receptacle flat or slightly convex, epaleate, ridges fringed with minute trichomes; florets ca. 17 in capitula, corollas lavender to reddish, with basal tube narrowly cylindrical below, throat short, lobes narrowly lanceolate, without obvious multiple longitudinal ducts; anther thecae and apical appendages bearing glandular dots, bases spurred, acute, not tailed; style without enlarged basal node or disk, with sweeping hairs extending strongly on to upper shaft. Achenes cylindric, ca. 2 mm long, 10–ribbed, short twin-hairs dense on ribs, glands in furrows, with scattered idioblasts amongst elongate surface cells, walls with sub-quadrate raphids; pappus of ca. 40 scabrid capillary bristles ca. 7 mm long, with outer series vestigial and bristleform. Pollen ca. 40–50 µ in diam. in fluid, tricolporate, sublophate with continuous perforated tectum between colpi.

######### Type material.

The single species is as follows: *Vernoniaincana* Less., Linnaea 4: 277. 1829. TYPE: “Brasilia meridionalis legit Sello[w]”, [between Rio Grande do Sul, São Gabriel and Uruguay, Catalán Area]: Friedrich Sello[w] 3379, *s.d.* [1826]. **Lectotype here designated: LL 00373309**; isosyntypes: **BR** 0000005536627, **GDC** G00327411, **HAL** 0114069, **K** 000497031, **K** 001066026 (a mixed sheet with a Tweedie s.n., and Gillies 154-2 collection also mounted with the Sello[w] collection), **P** 00682761 and **P** 00682762. None of the isosyntypes has the collecting number so they cannot be considered isolectotypes. A photograph of a specimen of this gathering from B is mounted and filed at **F** 0BN014552. There may be other isosyntypes that are not available on line [Images of type material cited above can be found online at JSTOR-Plants, continuously updated].

######### Synonomy.

*Vernoniaimmunis* Griseb., Symb. Fl. Argent. 163. 1879.

*Cacaliaimmunis* (Griseb.) Kuntze, Revis. Gen. Pl. 2: 970. 1891.

*Cacaliaincana* (Less.) Kuntze, Revis. Gen. Pl. 3(2): 138. 1898.

######### Remarks.

**Figure 4.**
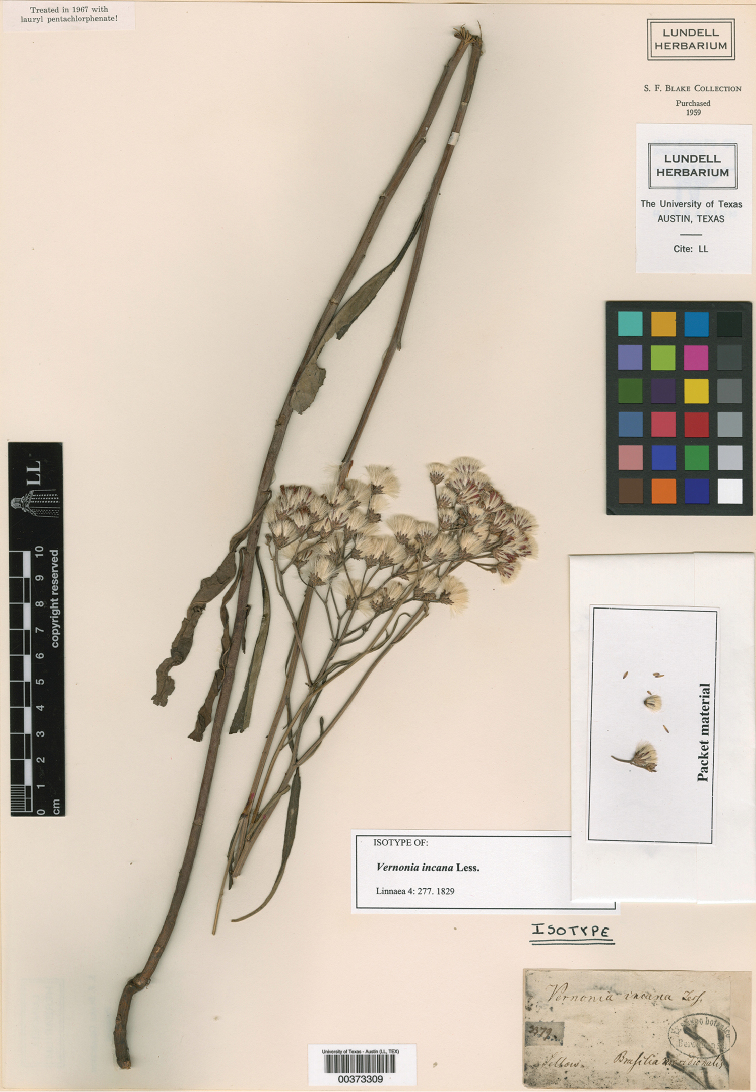
Image of the selected lectotype for *Vernoniaincana* Less. There are now six isosyntypes in European herbaria: all the specimens are similar, but the LL specimen was selected as the lectotype because it had the most complete information, including the collecting number and the label indicates that it had been in the Berlin herbarium before it was sent to S.F. Blake. [photo courtesy of JSTOR-Plants, continuously updated].

Lessing worked at Berlin (B) and all of the Compositae in that herbarium were destroyed during WWII (Hiepko 1987). There are seven isosyntypes in JSTOR from which one may select a Lectotype. There is also is a photograph at F of a B specimen that may have been some of the material Lessing studied but it is not indicated on the sheet. The LL specimen (now housed at TEX) was selected as the Lectotype (Fig. 4) because the label had the most complete information, including the collecting number and because the label information indicates that it had formerly been in the Berlin Herbarium. It was, at some point, sent to S.F. Blake (BARC) who worked at the herbarium of the US Department of Agriculture but also spent most of his free time at the US National Herbarium (US). In 1959, after his death, the Blake family sold his personal herbarium and library to C.L. Lundell (1959) who later transferred it to TEX [all of the Lundell specimens should be cited as LL]. The K specimen and one of the P specimens have a larger portion of the root than the others but they have less label information than the LL sheet. According to Vegter (1986), Friedrich Sello[w] lived from 1789 to 1831 and his original herbarium was at B. In TL II, Stafleu and Cowan (pg. 500–501, 1985) indicate that Sello changed the spelling to Sellow in 1914 but later his family dropped the ‘w’ and returned to the original spelling. It seems best to acknowledge the differing opinions and list him as Sello[w].

The collection date and location were determined by consulting Herter (1945) and Herter and Rambo (1953) which together provide a detailed guide to Sello[w]’s itinerary. Number 3379 was collected in 1826. The data from Herter (1945) and Herter and Rambo (1953) say that the numbers 3331–3623 were collected between Rio Grande do Sul, São Gabriel (Brazil) and Catalán, Uruguay. Given the low collecting number, it would seem fairly certain that this collection was made in 1826 in southern Brazil between São Gabriel in the state of Rio Grande do Sul (Brazil) and the Catalán area in the department of Artigas just south of the city of Artigas (Uruguáy) and just across the Uruguay River from Brazil. Both of the maps (Herter 1945 and Herter and Rambo 1953), however, show all 1826 collections as being south of the Uruguay river in what is now Uruguay. As a result, we are continuing to list the locality between Rio Grande do Sul, São Gabriel (Brazil) and Catalán, Uruguay.

According to J. Mauricio Bonifacino (MVFA), in the early 19^th^ Century, Brazil had control of Uruguay and some collections made at that time (from what is now southern Brazil and Uruguay) were labelled as “Brasilia meridionalis”.

## Supplementary Material

XML Treatment for Vernonia subg. Austrovernonia H.Rob. & V.A. Funk, subg. nov.
